# Biochemical and Structural Aspects of Cytokinin Biosynthesis and Degradation in Bacteria

**DOI:** 10.3390/microorganisms9061314

**Published:** 2021-06-16

**Authors:** Jitka Frébortová, Ivo Frébort

**Affiliations:** Centre of the Region Haná for Biotechnological and Agricultural Research, Czech Advanced Technology and Research Institute (CATRIN), Palacký University Olomouc, 783 71 Olomouc, Czech Republic; ivo.frebort@upol.cz

**Keywords:** cytokinin, isopentenyl transferase, tRNA modification, cytochrome P450 monooxygenase, LOG, CKX

## Abstract

It has been known for quite some time that cytokinins, hormones typical of plants, are also produced and metabolized in bacteria. Most bacteria can only form the tRNA-bound cytokinins, but there are examples of plant-associated bacteria, both pathogenic and beneficial, that actively synthesize cytokinins to interact with their host. Similar to plants, bacteria produce diverse cytokinin metabolites, employing corresponding metabolic pathways. The identification of genes encoding the enzymes involved in cytokinin biosynthesis and metabolism facilitated their detailed characterization based on both classical enzyme assays and structural approaches. This review summarizes the present knowledge on key enzymes involved in cytokinin biosynthesis, modifications, and degradation in bacteria, and discusses their catalytic properties in relation to the presence of specific amino acid residues and protein structure.

## 1. Introduction

Cytokinins (CKs) are a group of plant hormones that, together with other plant hormones, promote cytokinesis and affect many other aspects of plant development [1]. Despite the classification as plant hormones, CKs are evolutionally older than plants, constituting a class of highly conserved low-molecular-mass molecules present in many organisms, including bacteria, amoebae, filamentous fungi, algae, nematodes, insects, and humans. The evolutionally fixed function of CKs as a component of tRNA to serve mainly in improving the translation efficiency and fidelity developed into more specific roles of signaling molecules in plants and plant-interacting organisms, which often use CKs as tools for manipulating the plant host’s growth and development [2].

Naturally occurring CKs are adenine derivatives, with either isoprenoid or aromatic side-chain attached to the exocyclic nitrogen atom. These include *N*6-(2-isopentenyl) adenine (iP), *trans*-zeatin (tZ), *cis*-zeatin (cZ) and dihydrozeatin (DHZ) in the group of isoprenoid CKs, and benzyladenine (BA), its hydroxylated derivatives *ortho*- and *meta*-topolin (oT, mT) and their methoxy-derivatives as representatives of the aromatic CKs. The spectrum of CK molecules further extends to various conjugates with sugars, sugar phosphates, and amino acids (such as ribosides, ribonucleotides, *N*-glucosides, *O*-glucosides, *O*-xylosides, lupinic acid, or discadenine), or other compounds substituted either at the C2 position of adenine ring (2-methylthiolated CKs) or on the isopentenyl side-chain (mono- and dimethylated iP) [3,4]. These structural variations affect the biological activity of CKs and determine the function and compartmentalization of the respective CK metabolites [2]. Balance in the levels of CKs with different biological activities is then essential for individual plant developmental processes.

**Figure 1:**
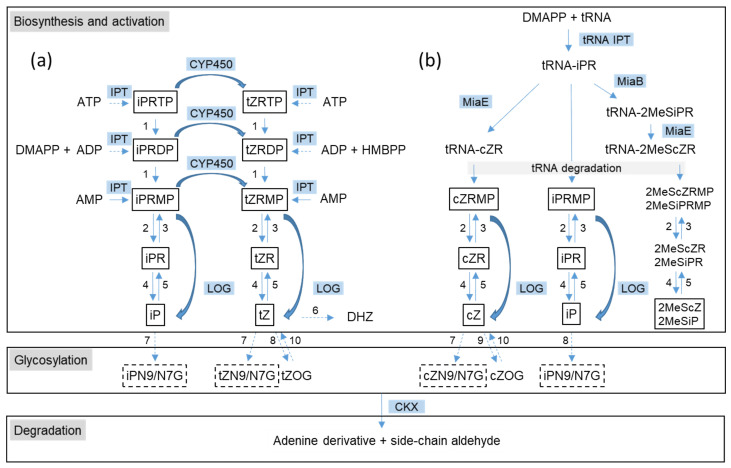
An overview of cytokinin (CK) metabolism as identified in plants and bacteria. (**a**) De novo CK biosynthesis pathway; (**b**) tRNA-dependent CK biosynthesis pathway. The key enzymes involved in CK biosynthesis and activation are isopentenyl transferase (IPT), cytochrome P450 monooxygenase (CYP450), CK phosphoribohydrolase “Lonely guy” (LOG), tRNA isopentenyl transferase (tRNA IPT), 2-methylthio-N6-dimethylallyladenosine synthase (MiaB), and tRNA 2-methylthio-N6-isopentenyl-adenosine(37) hydroxylase (MiaE). Other unspecific enzymes may be involved: phosphatase (1), 5′-ribonucleotide phosphohydrolase (2), adenosine kinase (3), adenosine nucleosidase (4), and purine nucleoside phosphorylase (5). tZ can be converted to dihydrozeatin (DHZ) by zeatin reductase (6). Deactivation of CKs is performed by CK *N*-glucosyltransferase (7), tZ-*O*-β-d-glucosyltransferase (8) and cZ-*O*-β-d-glucosyltransferase (9). *O*-glucosyl moiety can be removed by β-glucosidase (10). Irreversible degradation of CKs is catalyzed by CK dehydrogenase (CKX). Confirmed substrates of CKX are shown in boxes; only CK-*N*9 glucosides but not CK-N7 glucosides are CKX substrates (depicted by dashed boxes). CK abbreviations: iP, isopentenyladenine; iPR, isopentenyladenosine; iPRMP, iPRDP, iPRTP, isopentenyladenine riboside 5′-mono-, di-, triphosphate; tZ, *trans*-zeatin; cZ, *cis*-zeatin; ZR, zeatin riboside; ZRMP, ZRDP, ZRTP, zeatin riboside 5′-mono-, di-, triphosphate; 2MeSiPR, 2-methylthio isopentenyladenosine; 2MeScZR, 2-methylthio-*cis*-zeatin riboside; 2MeSiPRMP, 2-methylthio isopentenyladenine riboside 5′-monophosphate; 2MeScZRMP, 2-methylthio-*cis*-zeatin riboside 5′-monophosphate; iPN9/N7G, isopentenyladenine *N*9- or *N*7-glucoside; ZN9/N7G, zeatin *N*9- or *N*7-glucoside; ZOG, zeatin *O*-glucoside. The pathways involving the enzymes not described in bacteria are shown by dashed arrows. There is no experimental evidence for the presence of 2MeSCK riboside 5′-monophosphates in plant or bacterial samples, and their conversion to corresponding free bases by LOG has not been tested.

As indicated by the complexity of CK metabolites, various enzymes are involved in their biosynthesis and modifications, depending on the specific organism [3,5] (Figure 1). 

Biosynthesis of isoprenoid CKs starts with prenylation of N6-amino group of either free adenosine nucleotides (AMP, ADP, or ATP) or tRNA-bound adenosine phosphate in tRNAs recognizing the UNN codon. The reactions are catalyzed by two different classes of dimethylallyltransferases (commonly known as isopentenyl transferases, IPT): an adenylate IPT (EC 2.5.1.27 for AMP-dependent and EC 2.5.1.112 for ADP/ATP-dependent IPT, respectively) [6,7] and tRNA IPT (EC 2.5.1.75) [8]. The tRNA bound isopentenyladenine riboside (iPR) can be further modified by tRNA 2-methylthio-*N*6-dimethylallyladenosine synthase (EC 2.8.4.3) [9] at the C2 position of the adenine ring and by tRNA 2-methylthio-*N*6-isopentenyl-adenosine (37) hydroxylase (EC 1.14.99.69) [10] at the isopentenyl side-chain, which results in the formation of tRNA-bound 2-methylthio iPR (2MeSiPR), *cis*-zeatin riboside (cZR), and 2-methylthio-*c*is-zeatin riboside (2MeScZR). Free iP riboside-5′-mono-, di- and triphosphates (iPRMP, iPRDP, iPRTP) produced by adenylate IPT can be hydroxylated to corresponding tZ nucleotides by cytochrome P450 monooxygenase. CK riboside 5′- monophosphates liberated upon tRNA degradation or produced by adenylate IPT (with subsequent dephosphorylation in the case of CK riboside 5′- tri- and diphosphates) are converted to CK bases by CK-specific phosphoribohydrolase (EC 3.2.2.n1) [11] termed “lonely guy” (LOG). LOG enzyme also transforms both *trans*- and *cis*-ZRMP to their free bases. In addition to the LOG enzyme, enzymes of common purine metabolism, such as phosphatases, nucleotidases, nucleosidases, kinases, and phosporibosyl transferases, seem to be involved in a step-wise conversion of CK nucleotides to ribosides and free bases, as well as in the conversions in the opposite direction, as many of the enzymes catalyze reverse reactions. Phosphatases, nucleotidases, and nucleosidases are currently the only known enzymes producing CK ribosides; enzymes specifically converting either CK riboside 5′- monophosphates or CK bases to CK ribosides were not described. On the other hand, pathways leading to DHZ and aromatic CKs remain unclear: involvement of zeatin reductase (EC 1.3.1.69) in the conversion of zeatin to DHZ was only shown at the protein level [12] and biosynthesis of aromatic CKs is entirely unknown.

Deactivation of CKs proceeds through glycosylation by *N*- or *O*-glucosyltransferases. *N*-glucosides are mostly formed at the N7 and N9 position by *N*-glucosyltransferase (EC 2.4.1.118) [13], while *O*-glucosides are formed in CKs with a hydroxylated side-chain by *O*-β-d-glucosyltransferases, which can either transfer glucosyl moiety specifically to cZ (cZ-*O*-β-d-glucosyltransferases, EC 2.4.1.215) [14] or both cZ and tZ (tZ-*O*-β-d-glucosyltransferases, EC 2.4.1.203) [15]. The latter can also transfer xylosyl moiety; moreover, *O*-glucosyltransferases from various species may act on a different glycosyl group or use other hydroxylated CKs as substrates. The *O*-glycosylation is viewed as a reversible mode of CK deactivation as the *O*-glucosyl moiety can be removed by β-glucosidase (EC 3.2.1.21) [16]. *N*-glucosylation, on the other hand, is referred to as irreversible CK deactivation, although this view was recently questioned for tZ metabolism in *Arabidopis thaliana* [17]. Finally, complete degradation of CKs to adenine (or its N9- or C2-substituted derivatives) and a side-chain-derived aldehyde is achieved by side-chain cleavage catalyzed by CK dehydrogenase (CKX; EC 1.5.99.12) [18]. 

The survey of a spectrum of CK metabolites produced by plants and various microorganisms suggests that not all pathways involved in plant CK metabolism function in microorganisms. For example, there is no experimental evidence for the presence of aromatic CKs in bacteria. Cyanobacteria and microalgae generally lack CK-*N*-glucosides and often contain very low concentrations of zeatin-O-glucosides, tZ, and DHZ [19,20,21]. A similar distribution of CK bases was also observed in the plant pathogen *Rhodococcus fascians* [22], and low tZ levels compared to iP were found in the human pathogen *Mycobacterium tuberculosis* [23]. Some bacterial genera, however, produce tZ-type CKs as abundant or dominant species [24,25,26,27]. In addition to the missing or unrecognized *N*9-glucosylation pathway of CK deactivation, most bacteria also lack CKX to degrade CKs [28,29]. On the contrary, the only characterized CK species exclusive to bacteria are derivatives of iP with mono- and dimethylated isopentenyl side-chain (1-MeiP and 2-MeiP) [4]. In addition, the origin of cZR and 2MeScZR in plants must differ from that in bacteria due to the absence of the *miaE* gene in eukaryotes [5]. This review will focus on the structural and biochemical characterization of key proteins of CK metabolism present in bacteria.

## 2. The Initial Step of CK Biosynthesis: Two Different Pathways

Similar to plants, two different classes of IPTs, adenylate IPT and tRNA IPT, are involved in the initial step of CK biosynthesis in bacteria. The former transfers isopentenyl chain from dimethylallyl pyrophosphate (DMAPP) or 4-hydroxy-3-methyl-but-2-enyl pyrophosphate (HMBPP) to *N*6-amino group of AMP [3], while the latter uses the same side-chain donors to prenylate tRNA-bound adenosine phosphate in tRNAs recognizing the UNN codon [8]. In microorganisms, the tRNA-dependent production of CKs appears to play a dominant role. Many bacteria can produce CKs [30], but only a few of them contain adenylate IPT. Paradoxically, bacterial adenylate IPTs were characterized much earlier than those of plants.

### 2.1. De Novo CK Biosynthesis Pathway

Most described bacterial adenylate IPTs are associated with plant pathogens, such as *Agrobacterium tumefaciens* [31,32], *Pseudomonas savastonii* [33], *R. fascians* [22,34], *Ralstonia solanacearum* (former name *Pseudomonas solanacearum*) [35], *Pantoea aglomerans* (former name *Erwinia herbicola*) [36], or *Streptomyces turgidiscabies* [37]. Only recently, adenylate IPT from cyanobacterium *Nostoc* sp. PCC 7120, not involved in plant pathogenesis, was cloned and biochemically characterized [28]. The presence of an *IPT* gene with extremely high homology to *IPT* (*Tmr*) from some strains of *A. tumefaciens* was detected in two phototrophic non-sulfur purple bacteria *Rhodobacter sphaeroides* and *Rhodopseudomonas palustris* [38]. Several recent evolutionary analyses of *IPT* genes indicate the limited presence of adenylate IPTs among bacteria: in addition to earlier characterized IPTs, genes encoding IPT were identified only in *Anabaena variabilis* ATCC 29413, a cyanobacterial species closely related to *Nostoc* sp. PCC 7120, and the plant pathogenic bacteria *Agrobacterium vitis* and *Xathomonas albilineans* [28,39,40]. With few exceptions, the function of the genes as adenylate IPTs was assessed either by screening the release of CKs into culture media or predicted by sequence similarity to genes with established functions. 

The biochemical studies published on adenylate IPTs from different organisms indicate that bacterial IPTs prefer AMP as the prenyl chain acceptor and use both DMAPP and HMBPP as prenyl chain donors, while plant IPTs use DMAPP to prenylate preferentially ATP or ADP [3,22,28]. The use of HMBPP as a substrate allows for direct production of tZ-type CKs without the need for subsequent hydroxylation of iP-type CKs by P450 monooxygenase [41]. The first characterized genes encoding adenylate IPTs were those from *A. tumefaciens* [31,32,42], which contains two adenylate IPTs, tumor morphology rooty (Tmr), and tZ synthesizing (Tzs). Both encoding genes are located on Ti-plasmid. *Tmr* gene is only expressed after integration into the plant host chromosome following the infection, while *Tzs* is functioning in the bacterial cell. Characterization of the proteins showed that Tmr and Tzs both use AMP, DMAPP, and HMBPP as substrates (Table 1) to produce iPRMP and tZRMP, respectively [43,44,45].

The gene encoding adenylate IPT (*fasD*, also termed *fas4*) of *R. fascians* was identified as a part of the plasmid-located *fas* operon, consisting of six genes essential for virulence [34,46]. The protein was later expressed in *Escherichia coli*, purified, and shown to use both DMAPP and HMBPP as side-chain donors, with a preference for DMAPP [22] (Table 1). AMP was the preferred side-chain acceptor, but both AMP and ADP were prenylated by HMBPP, although with much lower efficiency in the case of ADP. In addition, as shown by Radhika et al. [4], FasD may also transfer monomethylated and dimethylated chains of isopentenyl pyrophosphate (IPP), yielding unique CK derivatives, 1-MeiP and 2-MeiP, which are the likely causative factors of the leafy gall disease [47]. The precursor molecules to the FasD catalyzed reaction, methylated IPPs, are produced by two methyltransferases (mt1 and mt2) located on the plasmid upstream of the *fas* operon. The preferred side-chain acceptor molecule and kinetic parameters of the reaction of methylated IPP with FasD are yet to be determined; it will be interesting to compare turnover rates for the common and newly discovered IPT substrates, respectively. 

The presence of a putative adenylate IPT gene in a non-pathogenic bacterium was first reported in 2003 [48]. The gene from cyanobacterium *Nostoc* sp. PCC 7120 (*NoIPT1*) was cloned and expressed in *E. coli* [28]. The recombinant enzyme was active with DMAPP and AMP, but not ADP and ATP as substrates (Table 1). HMBPP as a side-chain donor was utilized by the enzyme with a low reaction rate (less than 1% of DMAPP, which did not allow more detailed biochemical characterization), while IPP was not a substrate. The enzyme did not exhibit tRNA IPT activity either. The low reactivity with HMBPP resembles characteristics of IPTs of plant origin, which makes the enzyme dissimilar to other bacterial adenylate IPTs.

**Figure 2:**
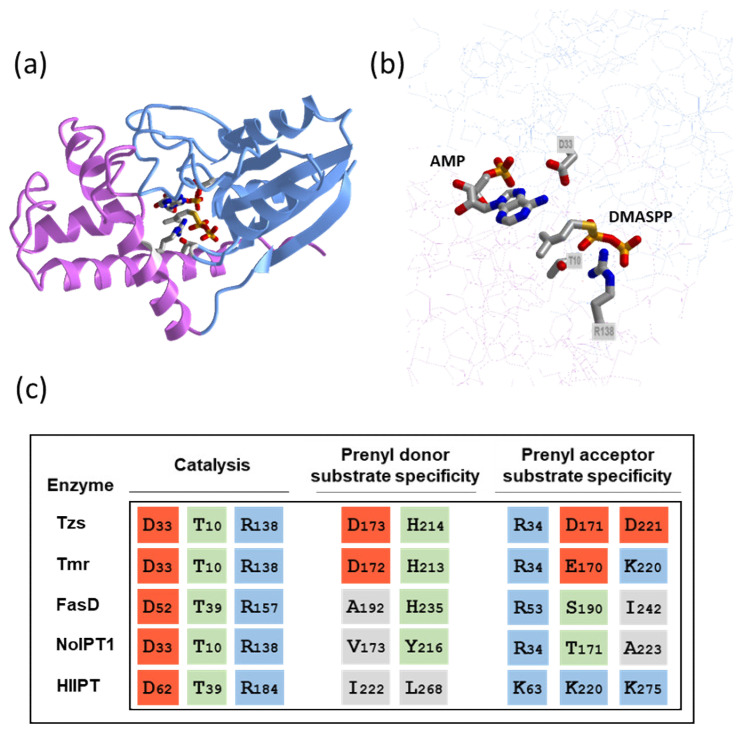
Structural insight into the function of adenylate IPTs. (**a**) Overall structure of Tzs from *Agrobacterium tumefaciens* in complex with AMP and donor substrate analog dimethylallyl S-thiodiphosphate (DMASPP). The *N*- and *C*-terminal domains are shown in light blue and magenta, respectively. (**b**) Close-up of the active site showing substrates and conserved residues involved in catalysis. The images were drawn by iCn3D structure viewer [50] using Protein Data Bank (PDB) coordinates 2ZE6 [45]. (**c**) Alignment of key amino acid residues in adenylate IPTs. The residues involved in enzyme catalysis and recognition of prenyl donor and prenyl acceptor substrates are depicted. Hydrophilic region formed by the side chains of histidine H214 and aspartic acid D173 determines the specificity of Tzs for HMBPP, while the arginine R34 interacts with α-phosphate group of AMP. Acidic, basic, polar, and hydrophobic residues are highlighted by red, blue, green, and grey backgrounds, respectively. Sequences of four biochemically characterized bacterial IPTs (Tzs, Tmr, FasD, and NoIPT1) are aligned with that of hop IPT (HlIPT) as a representative of plant IPT with known crystal structure.

Observed differences in substrate specificities of bacterial and plant IPTs can be explained by differences in protein structure. The crystal structure of Tzs protein together with site-directed mutagenesis of selected amino acid residues provided the first structural insight into the substrate recognition and reaction mechanism of adenylate IPTs, which was further extended by distinct features observed in the crystal structure of adenylate IPT from hop (*Humulus lupulus*, HlIPT) [45,49]. Both enzymes are composed of two domains, the interface of which forms a solvent-accessible reaction channel (Figure 2a). The *N*-terminal core regions are structurally related to that of the p-loop-containing nucleoside triphosphate hydrolase superfamily. The adenine group of adenosine phosphate is deeply buried inside the channel. The N6 position of adenosine phosphate interacts with the side-chain of a conserved Asp residue (Asp33 and Asp62 in Tzs and HlIPT, respectively; Figure 2b,c), which functions as a base to deprotonate the *N*6-amino group of AMP/ATP. The resulting nucleophile attacks the C1 carbon of DMAPP, which leads to the transfer of the isopentenyl group from DMAPP to accepting adenosine phosphate.

Two other conserved amino acid residues, Thr10 and Arg138, interact with the α-phosphate group of DMAPP and are necessary for efficient catalysis (Figure 2b,c) [45]. In the case of hop IPT, the β- and γ-phosphate groups of ATP in the active site interact with two basic residues Lys220 and Lys275. These are replaced by two acidic Asp residues (Asp171, Asp 221), suggesting that ATP or ADP cannot bind to Tzs due to repulsive forces between the phosphate groups and Asp. The third lysine residue of hop IPT interacting with β- and γ-phosphate groups (Lys63) is in Tzs replaced by Arg34, which forms a hydrogen bond with the α-phosphate group of AMP. Alignment of amino acid sequences of characterized bacterial adenylate IPTs with those of HlIPT and Tzs shows that the residues found critical to ATP binding in HlIPT differ in bacterial proteins. Lys63 of HlIPT is substituted by Arg in all bacterial sequences, while Lys220 is substituted by polar amino acids. Tmr of *A. tumefaciens* retains Lys at the position corresponding to Lys275, while NoIPT1 and FasD contain hydrophobic amino acids (Figure 2c).

Concerning the activity with prenyl donor substrate, His214 and Asp173 of Tzs, forming the hydrophilic region in the reaction cavity, are crucial for substrate recognition and specificity. Although mutant proteins were still able to react with HMBPP, both *K*m and *k*cat were strongly affected. In contrast, the changes in reactivity to DMAPP were small [45]. Both residues are also present in Tmr (His213 and Asp172), while FasD retains only one of these residues (His235) and the other is substituted by hydrophobic Ala192. NoIPT1 contains two substitutions, one amphipathic (Y216) and one hydrophobic (V173). Observed activities with DMAPP and HMBPP thus correspond to the presence or absence of two hydrophilic charged residues in the active site (Table 1, Figure 2c).

### 2.2. tRNA-Dependent CK Biosynthesis Pathway

In most bacteria, CKs are only found as nucleosides covalently incorporated into the tRNA sequence. The main function of isopentenyl modifications of tRNA is related to translation efficiency and fidelity, which consequently affects metabolic functions [8]. The isopentenyl-modified nucleotides are located in position 37 of the anticodon loop of tRNAs that read codons starting with U [8]. The first step leading to the synthesis of CK derivatives in tRNA is the isopentenylation of the exocyclic amine of A37 in tRNAs with adenosine in the third anticodon position (position 36) by tRNA IPT, resulting in the formation of iPR [8]. This is followed by methylthiolation, and/or, in some organisms, by *cis*-hydroxylation (for review, see [51]). The *miaA* genes encoding tRNA IPT are present in all bacterial species, with the exception of the genus *Mycoplasma* [39]. The high content of cZ-type and iP-type of CKs compared to the tZ-type in bacteria is attributed to tRNA degradation, which is the only confirmed pathway of cZ biosynthesis. In some bacterial species, e.g., from the genus of plant-growth-promoting bacteria *Methylobacterium* sp. or root nodule-forming *Bradyrhizobium* sp., tRNA degradation was assigned as a source of tZ [25,26]. Interestingly, most of the studied *Methylobacterium* species did not produce cZ metabolites and only some of them produced low levels of iP metabolites [27]. These observations could suggest that tRNA IPT preferentially uses HMBPP as a side-chain donor. The biochemical studies of tRNA IPTs are, however, hindered by the availability of the acceptor substrate, unmodified tRNA, as well as by complicated assay procedure. Nevertheless, the kinetic studies can be performed with chemically synthesized 17-base oligoribonucleotides, which mimic the stem-loop region of *E. coli* tRNA^Phe^ [52]. Using the consensus oligoribonucleotide [53], tRNA IPT from *Nostoc* sp. PCC 7120 (NoIPT2) was found to use both DMAPP and *trans*-HMBPP as donor substrates, with a strong preference for DMAPP [20].

tRNA sequences that are modified by bacterial tRNA IPTs share several highly conserved regions within the anticodon stem-loop, including an A36-A37-A38 motif, in which A37 is isopentenylated [52]. Site-directed mutagenesis of conserved amino acids in tRNA IPT of *E. coli* indicated amino acid residues involved in DMAPP binding (Lys23, Thr24, His67, and Arg217), tRNA binding (Lys56, Arg167, Arg170, and Lys280), and stabilization of developing positive charge in the transition state for electrophilic alkylation (Thr19 and Tyr47) [53].

**Figure 3:**
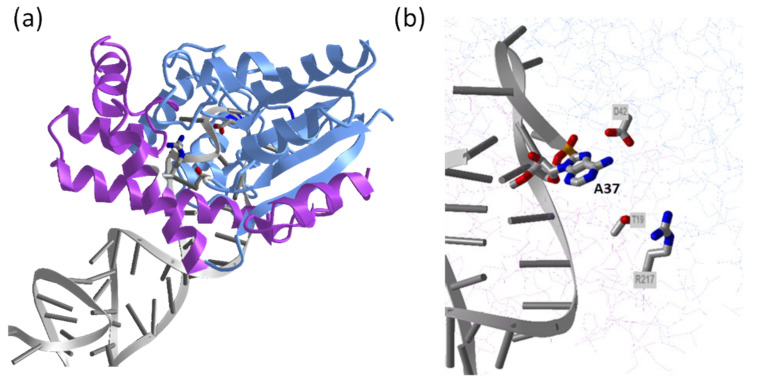
Structure of *Escherichia coli* tRNA IPT in complex with *E. coli* tRNA^(Phe)^. (**a**) Overall structure with the core domain shown in light blue, the insertion domain in magenta, and tRNA^(Phe)^ in grey. (**b**) Close-up of the active site showing adenosine substrate (A37) on tRNA anticodon loop and conserved residues Asp, Thr, and Arg (D42, T19, and R217, respectively) involved in the catalysis. The images were drawn by iCn3D structure viewer [50] using PDB coordinates 3FOZ [56].

The comparison of crystal structures of several tRNA IPTs as apo-form and in various complexes with substrates or their analogs [54,55,56] show that the tRNA IPT proteins are composed of two domains, a large core domain and a small insertion domain (Figure 3). The tRNA anticodon stem-loop binds between the two domains in a deep tRNA-binding cleft formed only upon tRNA binding. The cleft contains positively charged residues that pair with negatively charged tRNA. The binding of tRNA results in a conformational change in the anticodon loop, which partially unfolds. The target A37 nucleotide (together with several others) flips out and enters the channel in the core domain. This allows the other substrate, DMAPP, to enter the opposite side of the channel. The binding of DMAPP is not possible in the absence of tRNA substrate [54,57]. Despite the enormous difference in the structure of the side-chain accepting substrate, tRNA IPT and adenylate IPT share a conserved reaction mechanism involving matching amino acid residues. DMAPP substrate is in both IPT classes recognized by amino acid residues that are located within the p-loop close to the *N*-terminus, together with a conserved Arg residue. Similar to adenylate IPT, an aspartate residue (Asp42 and Asp33 in *E. coli* tRNA IPT and Tzs, respectively) is involved in the catalysis as a general base accepting a proton from the N6 atom of A37 in tRNA (Figure 2 and Figure 3). Nucleophilic attack on the carbon C1 of DMAPP then results in the transfer of dimethylallyl moiety from DMAPP to N6 atom of A37. Two other residues conserved in both tRNA IPT and adenylate IPT, Thr and Arg (Thr19 and Arg217 in *E. coli* tRNA IPT, corresponding to Thr10 and Arg138 in Tzs), interact with the bridging oxygen of DMAPP through hydrogen bonds and function to activate the transferring group and stabilize the leaving pyrophosphate group, respectively (Figure 2 and Figure 3) [54,55].

## 3. Formation of *Trans*-Zeatin: Unclarified Pathways

As described above, one of the pathways leading to tZ-type CKs is a direct transfer of HMBPP to AMP by adenylate IPT (so-called iPRMP-independent pathway). Based on the substrate specificity of adenylate IPTs, the pathway functions at least in some bacteria (Table 1). For *A. tumefaciens*, this pathway even represents a tool to produce high amounts of tZ in order to induce tumorigenesis in the infected plant [41]. The pathway was also assumed to serve for the overproduction of tZ by the bacteroid form of *Rhizobium* sp. IC3342, known to cause the leaf curl syndrome in pigeonpea, but the respective gene encoding an adenylate IPT was not found [58]. In the second known pathway, iPRMP-dependent, tZ-type CKs are formed by hydroxylation of iP-type CKs by cytochrome P450 monoxygenase of the *CYP735A* subfamily [59]. The enzyme was not purified to date due to its instability, but assays employing a microsomal fraction of yeast expressing CYP as an enzyme source can be used. The only biochemically characterized CYPs from *A. thaliana* hydroxylate iP nucleotides (with preference to iPRMP and iPRDP), producing only tZ, but not cZ nucleotides [59]. Homologous gene (*fasA*) was identified on the *fas* operon of *R. fascians* [46]. The protein was not characterized but analysis of CK content in *fasA* mutant clearly indicated the involvement of FasA in the hydroxylation of iP to tZ-type CKs [22].

In addition, some bacteria contain tZ-type CKs in their tRNA [25,26,60]. The simplest explanation for tZ presence would be a direct transfer of hydroxylated side-chain to A37 in tRNA by tRNA IPT, such as observed in *Nostoc* [20]. It is generally accepted that tRNA 2MeSiPR monooxygenase (MiaE), the only enzyme known to hydroxylate tRNA-bound iP, synthesizes 2MeScZRMP and cZRMP [10,51,61]. Yet, *trans*-selective hydroxylation of tRNA-bound iPR or 2MeSiPR by the same enzyme was reported by others [62,63]. The origin of zeatins in the tRNA of some organisms is entirely unclear, as the presence of the *miaE* gene is reportedly limited to a few bacterial genera [64]; however, up-to-date results of BLASTp searches (not shown) using MiaE from *Pseudomonas putida* [65] as a query suggests the far wider distribution of MiaE among bacteria than previously anticipated.

## 4. Cytokinin Activation: Removal of Phosphoribose by LOG

The direct production of biologically active CK free bases from corresponding CK nucleotides is catalyzed by CK riboside 5’-monophosphate phosphoribohydrolase, known as LOG [11]. Although originally identified in plants, genes with sequence similarity to LOG are also found in a number of other organisms, even in those that do not produce CKs by de novo biosynthetic pathway [11,66,67,68]. Most of these genes were, however, misannotated as lysine decarboxylases, due to the presence of the PGGxGTxxE motif conserved in thousands of putative proteins. Determination of crystal structure of LOG from filamentous fungus *Claviceps purpurea,* together with analysis of multiple protein sequences, allowed the identification of proteins with a possible LOG function [69].

The characterized plant enzymes hydrolyze CK mononucleotides but not CK di- and trinucleotides, CK nucleosides, or AMP [11,66]. *R. fascians* contains two LOG genes, *fasF* located on the *fas* operon of virulence plasmid and another gene located on the chromosome [22,70]. FasF protein was able to convert iPRMP, cZRMP, and tZRMP to their respective corresponding bases [22]. Interestingly, the *fas* operon is absent in one specific *R. fascians* strain (A21d2) and a gene chimera that encodes a fusion protein with IPT and LOG domains is present [71]. A similar chimeric gene encoding bifunctional protein with confirmed IPT and LOG activities was also found in fungal plant pathogen *C. purpurea*, in addition to another LOG gene [67]. Yet, another LOG homolog was found in the human pathogen *M. tuberculosis* and confirmed to be CK-specific; it efficiently hydrolyzed iPRMP while the reaction was slow with AMP [23]. Unfortunately, phosphoribohydrolase activity of several other bacterial LOGs was assayed only with AMP as substrate, thus leaving the question of their specificity towards CK substrates unanswered [68,72,73,74,75]. The secretion of iP into culture medium during simultaneous expression of *Corynebacterium glutamicum* LOG and IPT in *E. coli* however suggests its CK-activating function [68].

**Figure 4:**
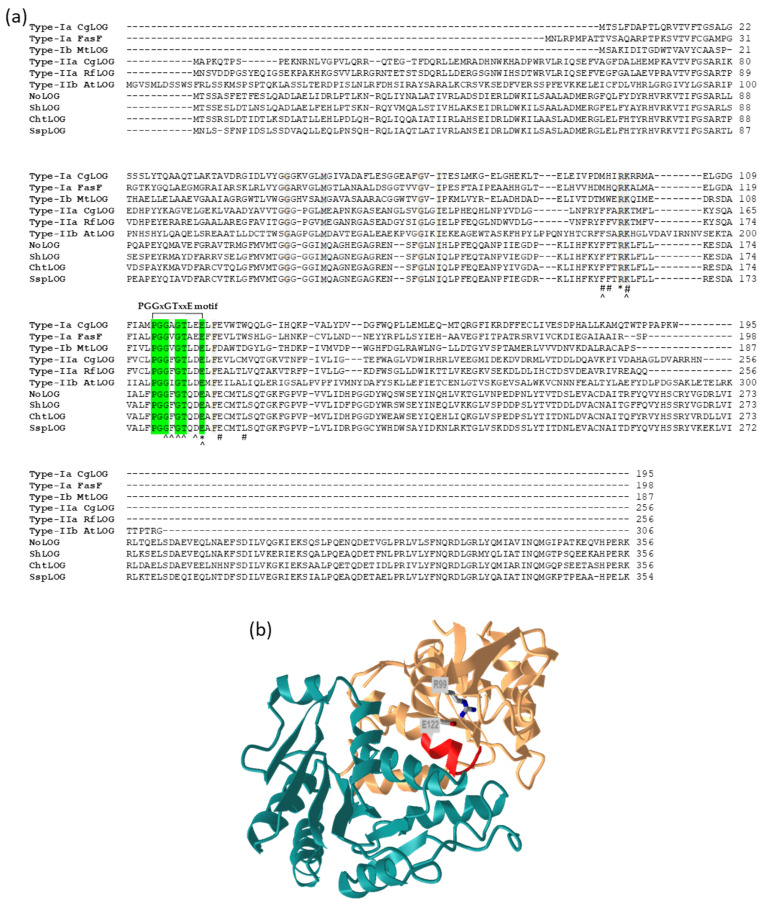
Amino acid composition and structure of LOG. (**a**) Amino acid sequence alignment of different LOG proteins. Protein sequences were aligned using the ClustalW interface in BioEdit 7.2.5 [77]. Conserved PDDxGTxxE motif is highlighted by green background and other fully conserved amino acid residues in grey. Residues involved in enzyme catalysis (*), AMP binding (^), and prenyl group binding (#) are indicated below the sequence [68]. Numbers on the right indicate an amino acid position in the respective protein. CgLOG, FasF, MtLOG, RfLOG, AtLOG, NoLOG, ShLOG, ChtLOG, and SspLOG are the abbreviations for LOGs from *Corynebacterium glutamicum* ATCC 13032, *Rhodococcus fascians* D188 (located on the virulence plasmid), *Mycobacterium tuberculosis* H37Rv, *R. fascians* D188 (located on chromosome), *Arabidopsis thaliana*, *Nostoc* sp. PCC 7120, *Scytonema hofmanni* PCC 7110, *Chroococcidiopsis thermalis* PCC 7203, and *Synechocystis* sp. PCC 7509, respectively. (**b**) Dimeric structure of a type I CgLOG showing the position of fully conserved catalytic residues Arg and Glu (R99 and E122, respectively) and PGGxGTxxE motif (in red) in one of the monomers. The two monomers are shown in gold and green, respectively. The image was drawn by iCn3D structure viewer [50] using PDB coordinates 5ITS [72].

Several classes of LOG enzymes were identified since their initial discovery, differing in the length of the *N*-terminal region and oligomeric state [68,75,76]. Based on the properties of characterized proteins and analysis of multiple protein sequences, the LOG proteins were classified as type I, containing dimeric proteins, and type II, containing hexameric proteins with an extended N-terminal region of about 50 to 60 amino acid residues. Both types can be further subdivided into two subgroups, based on variations in amino acids in the active site [68]. Type Ia LOGs include proteins from plants, *C. purpurea,* and some bacteria; type Ib and IIa contain bacterial proteins; and type IIb plant proteins. Some organisms (e.g., *C. glutamicum, R. fascians,* and *A. thaliana*) contain multiple LOGs belonging to different groups. In addition, some of the recently described LOGs cannot be classified into any of the existing groups [75,76]. One remarkable example is the LOG from the archaeon *Sulfolobus islandicus*, which forms a tetramer and contains a slightly modified GGGxGTxxE motif, which seems to be conserved not only in archaea but also in some thermophilic bacteria [76]. The search for proteins homologous to LOG in CK-producing *Nostoc* sp. PCC 7120 led to the identification of yet another type of putative LOG protein, which shows sequence characteristics representative of Type IIa LOG but contains extended *C*-terminal part of about 100 amino acid residues as compared to different LOG types (Figure 4a) [Frébortová, unpublished results]. The BLASTp search with *Nostoc* LOG protein (NoLOG) sequence as a query shows strong conservation of this long type LOG among cyanobacteria. Preliminary characterization of NoLOG expressed in *E. coli* shows that it is CK-specific phosphoribohydrolase converting iPRMP, tZRMP and cZRMP, but not their ribosides, to their corresponding free bases [Frébortová, unpublished results]. The oligomeric state and the function of the *C*-terminal domain are yet to be investigated.

Several LOG proteins, most of them of bacterial origin, were crystallized and their structure solved [68,69,72,73,74]. The first two structures described were those of dimeric LOGs, showing that dimerization creates a large substrate-binding pocket, in which two active sites containing PGGxGTxxE motifs are located (Figure 4b) [69,72]. The hexameric LOG proteins were shown to be composed of three dimers, in which the active site conformation is similar to those of dimeric LOGs [68]. The monomeric units of LOG proteins adopt an α/β Rossman fold with a differing number of α-helices and β-sheets. Based on the crystal structures and characterization of mutant proteins, two fully conserved amino acid residues, Arg and Glu, were proposed critical for catalytic activity (Figure 4). In addition, putative substrate-binding residues were described: the AMP moiety of nucleotide substrates is stabilized by conserved residues in the PGGxGTxxE motif, while the prenyl group binding residues are more variable, depending on LOG type (Figure 4a). Precise binding of the prenyl group is, however, uncertain, as no structure complexed with its native substrate is available. Solving the structure of LOG from *Pseudomonas aeruginosa* in its apo-form and complexed with AMP allowed the proposal of a catalytic mechanism for LOG, which involves the formation of oxocarbenium ion-like transition state [73].

## 5. Degradation of Cytokinins by Cytokinin Dehydrogenase

**Figure 5:**
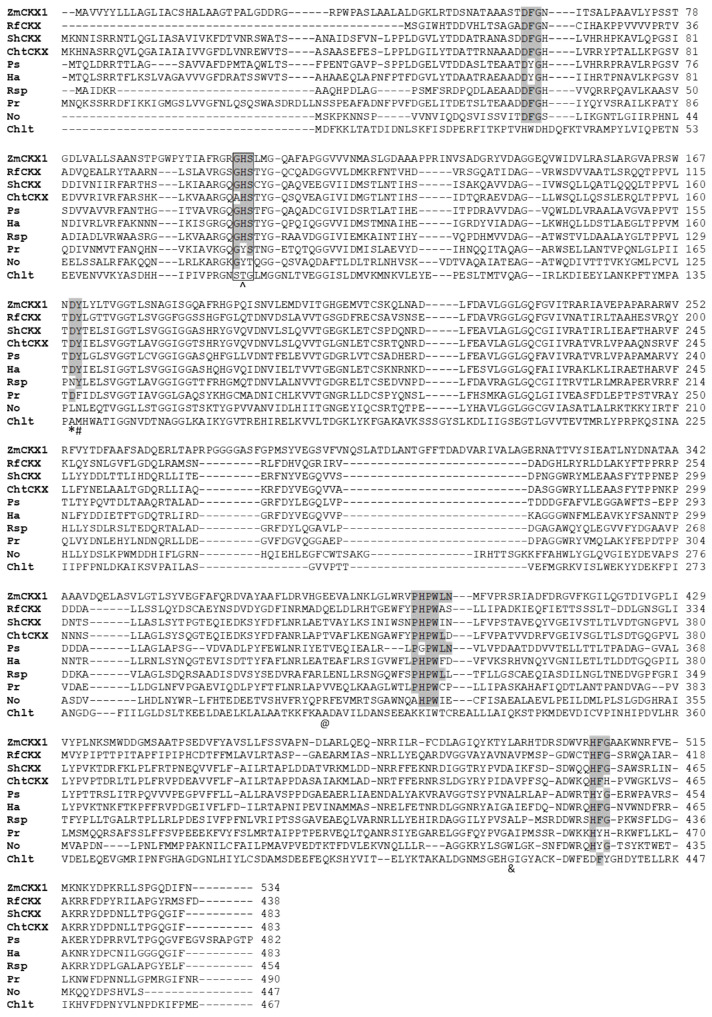
Amino acid sequence alignment of selected CKX and CKX-like proteins. Protein sequences were aligned using the ClustalW interface in BioEdit 7.2.5 [77]. Residues forming highly conserved motifs are highlighted in grey. The FAD-binding GHS motif is framed and the His residue forming the covalent bond with FAD is marked (^). Residues involved in enzyme catalysis (*), covalent flavinylation (#), reactivity with N9 glucosides (@), and cZ (&) are indicated below the sequence. Numbers on the right indicate amino acid position in the respective protein. The selection of bacterial proteins is based on evolutionary analysis of CKXs by Wang et al. [29]. ZmCKX1, RfCKX, ShCKX, and ChtCKX are the abbreviations for proteins with confirmed CKX activity from the following organisms: maize (reference plant CKX of known crystal structure), *Rhodococcus fascians* D188 (Actinobacteria), *Scytonema hofmanni* PCC 7110 (Cyanobacteria), and *Chroococcidiopsis thermalis* PCC 7203 (Cyanobacteria), respectively. Other included CKX-like proteins are Ps from *Promicromonospora sukumoe* (Actinobacteria), Ha, *Herpetosiphon aurantiacus* DSM 785 (Chloroflexi), Rsp, *Rhizobium* sp. YS-1r (Proteobacteria), Pr, *Pseudoalteromonas rubra* (Proteobacteria), No, *Nostoc* sp. PCC 7120 (Cyanobacteria), and Chlt, *Chlamydia trachomatis* (Chlamydia).

CKX is a key enzyme regulating the concentration of active CKs by irreversible, oxidative cleavage of CK side-chain [78]. It belongs to flavoprotein oxidoreductases from the vanillyl alcohol oxidase family containing covalently bound FAD cofactor [79,80]. While plant CKXs were extensively studied at the biochemical as well as a physiological level [5,81], *R. fascians* CKX (RfCKX or FasE) remains the only characterized functional CKX of bacterial origin [22]. The *CKX* gene of *R. fascians* was found on *fas* operon in 1994 [46] but its putative function in CK degradation was only revealed after the first *CKX* gene from maize (*ZmCKX1*) was cloned and the encoded protein characterized as CKX [82,83]. CKX-like sequences were later found in cyanobacterium *Nostoc* sp. PCC 7120 and two other cyanobacteria, *Synechocystis* sp. PCC 6803 and *Prochlorococcus marinus* [84]. The presence of CKX homologs was recently identified in about 2.5% of available bacterial proteomes and genomes within the evolutionary study of CKXs [29]. CKX-like genes were found mainly in the phyla Actinobacteria, Proteobacteria, and Cyanobacteria, with a few species belonging to the phyla Chlamydia and Chloroflexi. The cyanobacterial sequences included those shown earlier to lack several conserved residues indispensable for cofactor binding and catalysis, such as from *Acaryochloris marina*, *Nodularia spumigena*, and *Anabaena variabilis* [5]. Several conserved residues are absent in putative CKX protein sequences from various bacteria (Figure 5), which makes their function in CK degradation uncertain or even excluded. The crystal structure of ZmCKX1 and characterization of mutant proteins both clearly show that conserved Asp residue is essential for catalysis by polarizing N6 atom of the amino group of CK substrate facilitating hydride transfer to the N5 atom of FAD cofactor [80,85]. This catalytic Asp residue is substituted by hydrophobic Leu residue in putative CKX from *Nostoc* sp. PCC 7120 (NoCKX), which was shown to be inactive [28]. In addition, the FAD cofactor is in all active CKX proteins covalently bound to His residue within the conserved GHS motif in the *N*-terminal part of protein [5,80]. Mutation of this residue prevents covalent FAD binding and leads to a loss of protein structural integrity [85]. Although the *N*-terminal domain of NoCKX was identified as a FAD-binding domain by conserved domain search, NoCKX does not contain FAD, presumably due to the absence of the GHS motif (contains GYT motif instead) [28]. Even the replacement of Tyr residue by canonical His residue together with the simultaneous introduction of catalytic Asp did not restore FAD binding, which is in line with the finding that self-catalytic covalent flavinylation requires a Tyr residue adjacent to catalytic Asp residue which, however, is absent in NoCKX [28,86]. 

Examination of cyanobacterial CKX-like protein sequences indicates that some of them contain most or all of the important conserved domains, such as N-terminal DFG and GHS motifs, catalytic Asp residue, or C-terminal PHPWLN and HFG motifs [84,87] (Figure 5). Indeed, CKX activity was detected in cell lysates, when the selected proteins were expressed in *E. coli* [Frébortová, unpublished results]. Purified CKX from *Scytonema hofmanni* PCC 7110 (ShCKX) was able to cleave various CKs, including free bases, ribosides, riboside 5’- monophosphates, and *N*9-glucosides of iP, cZ, and tZ-type CKs. Remarkably, Ala residue replaces Gly residue in the GHS motif in the protein from *Chroococcidiopsis thermalis* PCC 7203. The protein is nevertheless active, but its activity is gradually lost during the purification procedure, indicating that the FAD cofactor is not covalently bound to the protein [Frébortová, unpublished results].

**Figure 6:**
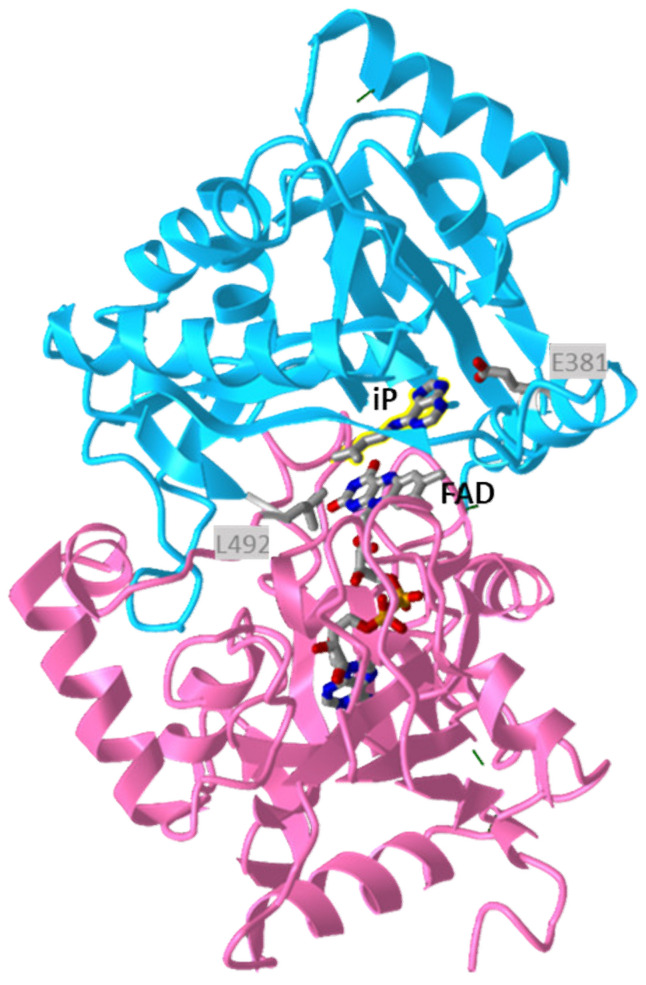
Structure of maize CKX (ZmCKX1) in complex with isopentenyladenine. The FAD-binding domain is shown in deep pink and the cytokinin-binding domain in deep sky blue. The FAD cofactor and iP substrate (highlighted in yellow) are shown, together with the Glu and Leu residues (E381 and L492, respectively), affecting the substrate specificity. The image was drawn by iCn3D structure viewer [50] using PDB coordinates 1W1Q [80].

The crystal structures of several plant CKXs [80,85,88] were solved, all showing the two-domain composition characteristic of oxidoreductases from the vanillyl alcohol oxidase family, consisting of FAD cofactor binding domain and substrate-binding domain (Figure 6). The CK adenine moiety binds in a funnel-shaped region on the protein surface, which is by a narrow pore connected to the internal cavity lined by the isoalloxazine ring of FAD. The pore is occupied by the C-N bond to be cleaved and the CK side-chain extends to the internal cavity. The cavity can accommodate both isoprenoid and aromatic side-chain [80], but all characterized CKXs show high activity with isoprenoid CKs and negligible activity with aromatic CKs, e.g., [89,90]. Various CKX isoforms exhibit different substrate specificities with individual CKs, which are determined by the presence of specific amino acids in the active site [85,90]. A key amino acid residue affecting the reactivity with N9 substituted substrates is the Glu residue at the entrance to the active site (Glu381 in ZmCKX1, Figure 5 and Figure 6), which decreases the affinity to CK ribosides and CK N9 glucosides and prevents efficient reaction with N9 glucosides by restricting ligand binding [85]. It was also shown that replacement of well-conserved Leu residue (Leu492 in ZmCKX1) located in the internal part of the substrate cavity (Figure 6) by smaller Ala improves reactivity with cZ and aromatic CKs. Not much is known about CKXs of bacterial origin, except that RfCKX containing Gln and Ala in corresponding positions degraded iP, iPR, iPRMP, and 2MeSiP with similar rates, showed low activity with iP9G, and higher activity with cZ derivatives than with tZ derivatives [22]. In addition, the broad substrate specificity observed with ShCKX corresponds to the absence of Glu residue and the presence of Ala residue in the active site (Figure 5). 

Upon oxidation of the CK substrate, the FAD cofactor is fully reduced to FADH_2_. The reduced cofactor is then re-oxidized either by quinones and other redox-active organic compounds acting as electron acceptors (dehydrogenase reaction) or oxygen (oxidase reaction). The reaction with oxygen proceeds with very low reaction rates [89,90]. The relative activity with oxygen seems to be determined by the presence of catalytic Asp residue and C-terminal Leu residue already implied in cZ reactivity: replacement of either of the two residues enhances the reaction rate with oxygen as electron acceptor relative to other compounds [85]. RfCKX, which contains Ala in the position corresponding to Leu of ZmCKX1 and many other plant CKXs, indeed uses oxygen rather efficiently, although the activity is higher in the dehydrogenase mode [22].

## 6. Conclusions

It has been known for several decades that bacteria can synthesize CKs [91], but most of them only in a form bound to tRNA. Notably, CKs are produced by plant-associated bacteria, both pathogenic and beneficial [92]. Production of CKs by plant pathogenic bacteria results in profound changes in plant growth such as the formation of the crown and leafy galls [93] and represents one of the strategies how plant pathogens benefit from the interaction with plant, i.e., hijacking hormone homeostasis to enhance nutrient acquisition [94]. The two best-known examples of plant pathogens with this strategy are *R. fascians* and *A. tumefaciens*. The actinomycete *R. fascians* contains a complete CK biosynthesis pathway, including IPT, CYP450, and LOG as a part of the *fas* operon, which is necessary for virulence [22,46]. Although it has been suggested that the pathology is induced by the secretion of a mix of six synergistically acting CKs [22,95], more recent studies support the involvement of newly discovered CK 2-MeiP produced only by virulent *R. fascians* strains in the development of disease symptoms [4,96]. Gene encoding active CKX (*fasE*) is also present in the *fas* operon, but it does not have a known function in pathogenesis and appears to be superfluous [71]. The Gram-negative gall-forming bacterium *A. tumefaciens* contains two IPT genes, *Tmr* and *Tz*s, both located on the tumor-inducing plasmid. During plant infection, the *Tmr* gene located in the T-DNA region is transferred to the host nuclear genome and functions in the host, while *Tzs* located in the virulence region of nopaline-type plasmid functions in the bacterium [3]. CKs produced by Tmr together with another plant hormone auxin induce gall formation [97], while CKs produced by Tzs regulate *vir* gene expression, thus affecting bacterial growth and virulence during infection [98].

Many rhizhospheric and epiphytic plant growth-promoting bacteria belonging to the diverse genera, including *Azospirillum*, *Bacillus, Pseudomonas, Rhizobium,* or *Methylobacterium*, can produce CKs which affect the growth and productivity of plants, increase tolerance to environmental stress, or enhance disease resistance [99,100,101]. CKs are also produced by various cyanobacteria [19,28,99,102]. Most studies are based on the observed secretion of CKs into the environment without analysis of corresponding genes and proteins. Given the limited presence of adenylate IPTs and the ubiquitous presence of tRNA IPTs in bacteria, it can be assumed that the majority of bacterially produced CKs originate from tRNA decomposition. The resulting CK monophosphates can then be directly converted to their respective CK bases as catalyzed by the ubiquitous LOGs.

In addition to bacteria involved in plant-microbe interaction, CK production was also reported in the human pathogen *M. tuberculosis*, which secretes several CKs [23]. The CK breakdown product, the side-chain aldehyde, is probably responsible for the increased sensitivity of the bacterium to nitric oxide. In mutants of this bacteria with inactivated proteasomal degradation, the LOG protein can accumulate, resulting in higher levels of active CKs. LOG levels are tightly controlled by the proteasome, suggesting that the bacteria could fine-tune CK production. It was hypothesized that CKs could either be used as signaling molecules to communicate among mycobacteria or to act on the host in order to facilitate infection [23]. More recent work demonstrated that CKs induce transcription of the mycobacterial gene of unknown function which results in a loss of characteristic acid-fast staining of *M. tuberculosis* [103]. Proteasomal regulation of CK activation by LOG is also involved in the mycobacterial resistance against antimicrobial antifolate drugs [104]. LOG proteins from other human pathogens *Pseudomonas aeruginosa* and *Bordetella pertussis* were also described [73,75]. The latter has wide substrate specificity to purine and pyrimidine monophosphates, producing 6-*O*-methylguanine as a physiological product, which sensitizes *B. pertussis* to oxidative stress [75]. The discovery of LOGs and their function in several unrelated human pathogens thus indicates that CKs have roles beyond plant development and plant-microbe interactions.

## Figures and Tables

**Table 1:** Kinetic parameters of bacterial isopentenyl transferases.

Enzyme	Tested Substrate	Second Substrate	*K*m (µM)	*k*cat (s^−1^)	Reference
Tmr	AMP	DMAPP	0.086 ± 0.008	4.1 × 10^−1^	[43]
	DMAPP	AMP	8.28 ± 0.82	n.a.	[43]
	DMAPP	AMP	10.1 ± 0.5	n.a.	[41]
	HMBPP	AMP	13.6 ± 2.5	n.a.	[41]
Tzs	AMP	DMAPP	0.035 ± 0.005	n.a.	[45]
	DMAPP	AMP	7.9 ± 0.6	5.3 × 10^−2^	[45]
	HMBPP	AMP	8.2 ± 0.4	2.5 × 10^−2^	[45]
FasD	DMAPP	AMP	0.030 ± 0.016	7.9 × 10^−3^	[22]
	HMBPP	AMP	0.026 ± 0.003	6.8 × 10^−4^	[22]
	HMBPP	ADP	0.550 ± 0.020	9.4 × 10^−4^	[22]
NoIPT1	AMP	DMAPP	0.63 ± 0.180	n.a.	[28]
	DMAPP	AMP	27.1 ± 2.47	5.3 × 10^−3^ *	[28]
	HMBPP	AMP	n.d.	n.d.	[28]

The Michaelis (*K*m) and catalytic rate constants (*k*cat) as determined for the tested substrate in combination with the second subScheme 1. for IPT from *Nostoc* sp. PCC 7120. Abbreviation: n.a. not available; n.d. not determined due to low reaction rate; * estimated from specific activity with 100 µM substrates.

## Data Availability

Data is contained within the article.
